# Use of herbarium data to evaluate weediness in five congeners

**DOI:** 10.1093/aobpla/plv144

**Published:** 2015-12-15

**Authors:** Ana M. Hanan-A., Heike Vibrans, N. Ivalú Cacho, José L. Villaseñor, Enrique Ortiz, Vinicio A. Gómez-G.

**Affiliations:** 1 Posgrado en Ciencias Biológico Agropecuarias, Universidad Autónoma de Nayarit, Carretera Tepic-Compostela Km 9, Xalisco 63780, Nayarit, Mexico; 2 Postgrado en Botánica, Colegio de Postgraduados, 56230 Montecillo, Texcoco, Estado de México, Mexico; 3 Instituto de Biología, Universidad Nacional Autónoma de México, Mexico City 04510, Mexico; 4 Facultad de Ciencias, Universidad Nacional Autónoma de México, Mexico City 04510, Mexico

**Keywords:** Distribution modelling, disturbed habitat, herbarium specimen data, MaxEnt, ruderal plants, synanthropy, weed, weediness index

## Abstract

It is often desirable to quantify a plant's relative weediness or synanthropy, that is, the degree to which a species associates with human-caused disturbance, in order to study and understand the biology, ecology and evolution of weeds and invasive plants. Herbarium specimens are among the most accessible and verifiable sources of data on distribution and habitat. However, the habitat distribution of species may not be reflected accurately by herbarium specimen data, due to well-known biases in plant collection. Here, we assess how well herbarium specimens reflect species' weediness, when compared with direct field surveys. We used five species of *Melampodium* (Asteraceae) and classified their degree of weediness with a modification of Nuorteva's synanthropy index, based on herbarium specimens. We then modelled the distribution of our focal species in Mexico using MaxEnt and identified a polygon of ∼3000 km^2^ in the state of Nayarit, Mexico, where there was a high probability of finding all five species. Systematic field searches in the target area documented all visible populations of four species along major and minor roads. Then we, again, classified their degree of weediness with the synanthropy index, based now on field data, and compared. We found that herbarium data were an accurate predictor of a species' weediness relative to its congeners despite the well-documented skew of herbarium data towards natural areas, which our data reflected as well. So, herbarium data can be used to classify species' weediness relative to each other, but not in absolute terms, if the specimens were correctly identified and none of the species were subject to particular collection bias. This study is the first attempt to compare herbarium and field data on this subject and may be relevant for other types of investigations based on herbarium data. Our work also highlights the usefulness of distribution models based on herbarium specimens.

## Abstract (precis)

A weed or not a weed? Many plant species grow somewhere on the continuum from undisturbed to very disturbed vegetation. Deciding on the degree of weediness is not an easy task, and often based only on subjective observations. In this work, we compare data obtained during systematic field surveys with the habitats recorded on herbarium specimen labels, for a group of more-or-less weedy tropical species. We show that herbarium data reflect the collection bias favouring natural vegetation, but also, that the relative weediness hierarchy stays in place. The study is relevant for other ecological studies based on herbarium specimens.

## Introduction

Weeds are plants that grow and form self-sustained populations in habitats strongly modified by humans (Harper 1944, cited in Baker 1965; Radosevich *et al*. 1997; Rzedowski 2006). They are often unwanted, but can also play a major role in landscape ecology and aesthetics, or form part of agricultural production (Vieyra-Odilon and Vibrans 2001). Because of their numerous interactions with humans, their biology, ecology, evolution and communities are an important subject of study (Kuester *et al*. 2014). They overlap partly with invasive plants, understood as introduced species that cause problems, either in agriculture or in natural areas.

Weediness or synanthropy, that is the degree to which a species associates with anthropogenic habitats, is not a binary trait, but one with many intermediates. However, it is often discussed in the literature as if it were, and without providing quantitative support for the assertion. Also, ranking species on a relative scale may be useful for comparative studies of the biological traits tied to weediness or invasiveness, for example.

Previous attempts have classified species according to their association with disturbance types or other species. For example, weedy species may be categorized based on attributes such as the ability to establish new populations in sites where the vegetation cover was removed (Hill *et al*. 2002) or the proportion of annual or introduced species in the vicinity of the species of interest (Hill *et al*. 2002). Degree and kind of disturbance of the habitats occupied by weeds have been the focus of other efforts. The degree of association of a plant species with human settlements (Klotz and Kühn 2002), or the average vegetation cover of urban soil associated with a species (Hill *et al*. 2002), can be documented. Frequently, these various metrics are calculated with the information obtained from raw data on species' occurrences available in public databases (e.g. data from phytosociological relevés of the Braun-Blanquet tradition).

Recent years have seen a strong increase in the use of data derived from biological collections as a primary and verifiable source to address diverse environmental and ecological questions (Graham *et al*. 2004; Bolmgren and Lönnberg 2005; Wu *et al*. 2005; Pyke and Ehrlich 2010; Robbirt *et al*. 2011; Lavoie 2013). Information on species habitat can be obtained from collections or from data obtained directly in the field. Both types of data have potential shortcomings.

When working with collections, it is important that identifications are corroborated and that labels are checked to identify obvious mistakes. Also, collections are often biased: biologists tend to collect in less disturbed, but accessible areas (Rich and Woodruff 1992; Kadmon *et al*. 2004; Crawford and Hoagland 2009; Kramer-Schadt *et al*. 2013); prefer rare over common species (Guralnick and Van Cleve 2005; Garcillán and Ezcurra 2011) and do field work during holidays and vacations (Pyke and Ehrlich 2010).

Field data are generally more accurate, but gathering them is time consuming, costly and usually limited in space and time. Guiding fieldwork with predictive modelling can help ameliorate these issues and make the process more efficient (Phillips *et al*. 2006; Mateo *et al*. 2011; Wegier *et al*. 2011; Gil and Lobo 2012). There are surprisingly few studies comparing the two types of data directly (e.g. Garcillán and Ezcurra 2011), and none for the group of species known as weeds.

When detailed data on the associated vegetation or disturbance type are not directly available, other sources have to provide data for the indicators. For example, the association of species with general habitat types rated by disturbance level, and the proportion of records per species and habitat can be calculated. The anthropogenic habitats populated by weeds vary in type, intensity and frequency of disturbance (Šilc 2010). One classification of weeds distinguishes plants that have to adapt to regular episodes of soil disturbance and removal of all aboveground biomass (agrestal weeds) from those that do not or for which disturbance is irregular (ruderal weeds) (Holzner 1978, 1982). For ruderal weeds, which often grow in or near human settlements, mowing, grazing or trampling may be the crucial disturbance types. Because soil and roots are not regularly disturbed, ruderal habitats are, on average, less stressful than agrestal sites.

Here, we assess whether herbarium specimens provide accurate weediness or synanthropy rankings, compared with field data. As a case study, we used five species of the genus *Melampodium* (Asteraceae) with differing degrees of weediness, judging from preliminary observations and data from floras. To assess weediness, we used a modified Nuorteva (1963) synanthropy index (SI) for insects, a metric that reflects the relative frequency with which a species is found in habitats disturbed to varying degrees. We followed Hart (1976) in deriving data from herbarium specimens. Species distribution models guided our field surveys; we searched for and documented populations and their habitats during peak flowering time. The modified Nourteva index was calculated for every species, first for the herbarium records, then for the field data. If herbarium data reflect relative weediness despite collection bias, then data obtained from specimens are a more accessible data source for assessing this characteristic than direct field surveys.

## Methods

### The genus *Melampodium*

*Melampodium* (Asteraceae) is a genus of ∼40 species, most of them annual, originally restricted to the tropical and subtropical regions of Mexico and Central America (Stuessy 1972; Blöch *et al*. 2009). The taxonomy and phylogeny of *Melampodium* are well known (Robinson 1901; Stuessy 1971, 1972, 1979; Stuessy *et al*. 2004; Blöch *et al*. 2009; Weiss-Schneeweiss *et al*. 2009; Blöch 2010). *Melampodium* is an appropriate system for the study of weediness as it has been widely collected in Mexico, and expert taxonomists specializing in the genus or the family (T. Stuessy and J.L.V., respectively) have examined the specimens. Moreover, ruderal populations are known for all species of the genus and agrestal populations for a third of the species. All but one species are annual; *Melampodium americanum* is considered perennial though its populations in Nayarit show a tendency towards an annual habit (Stuessy 1972). Phylogeny, karyotypes and allopolyploidy events are well documented (Stuessy 1971, 1972, 1979; Stuessy *et al*. 2004; Blöch *et al*. 2009; Weiss-Schneeweiss *et al*. 2009; Blöch 2010).

### The study area

We limited our field study to the state of Nayarit, on the western coast of Mexico. This state is located at the confluence of four Mexican physiographic regions: the Trans-Mexican Volcanic Belt, the western and the southern Sierra Madre mountain chains and the north-western coastal plain. It, thus, has a highly diverse flora (3428 species) that assembles into many vegetation types (Téllez 1995; Villaseñor 2003). Fourteen species of *Melampodium* have been reported for Nayarit (Stuessy 1972; McVaugh 1984; Villaseñor and Espinosa-García 1988; Téllez 1995; Ortiz *et al*. 1998), representing over half of the sections in the genus recognized by Stuessy (1972) and Blöch (2010). Also, over 60 % of the known range of the narrowly distributed *M. tepicense* is found in the state of Nayarit. Documented human settlements for the state date to prehispanic times (Anguiano 1992), allowing for varied associations between plants and human activities.

### Species selection and overview

Based on herbarium records and a preliminary field survey in October and November of 2011, we selected 5 species of *Melampodium*, all native, of the 14 species reported for Nayarit (Stuessy 1972; Ortiz *et al*. 1998): *M. americanum*, *M. divaricatum*, *M. microcephalum*, *M. perfoliatum* and *M. tepicense*. We aimed to cover as wide a range of weediness as possible. The general distribution, habitat information and phenology of the species included in this study and reported in the literature are shown in **Supporting Information—Table S1**.

We obtained the herbarium data from a national database maintained and curated by J.L.V., Asteraceae specialist and one of the authors of this study. It includes the herbarium specimens available at the National Herbarium (MEXU), with a few other additions. The database contained 1562 records with geographic coordinates for our five focal species. We modelled the potential distribution for each species using records from all of Mexico. By including data for as wide a portion of the species ranges as possible (and not only from the state of Nayarit; see below for the description of our distribution modelling approach), we hoped to capture more accurately the conditions under which the species occur, and thus increase the predictive power of our distribution models. Then, using our species distribution models as a guide for Nayarit, we conducted field surveys to locate populations and record population habitat at peak flowering time.

### Herbarium specimen data and habitat categories

We examined all specimens of our five focal species of *Melampodium* available at MEXU to verify their identification and information on their habitat. Habitat information was only available for 543 records, all of which we included in our study. The habitat data on the herbarium labels were assigned to three categories: (i) ‘natural vegetation’, if the reported habitat consisted of natural vegetation, even if disturbed, unless the plant was clearly part of secondary vegetation; (ii) ‘ruderal vegetation’, if the label described the habitat as ‘roadside’, ‘railroads’, ‘field margins’, ‘football fields’, ‘parking lots’, ‘old fields’, ‘pastures’, ‘secondary grassland’ or more generally, secondary vegetation and (iii) ‘agrestal vegetation’, if the collection was from cultivated fields, plantations or gardens.

### Distribution modelling

The national database contained 1562 records with geographic coordinates of our five focal species. Of these, 110 were of *M. americanum*, 935 of *M. divaricatum*, 142 of *M. microcephalum*, 347 of *M. perfoliatum* and 28 of *M. tepicense*. We randomly selected 75 % of these records to model the potential distribution of each species and set aside the remaining 25 % for verification of our models. We built our species distribution models with the program MaxEnt v. 3.3.3e (Phillips *et al*. 2006; http://www.cs.princeton.edu/~schapire/maxent/). As predictor variables, we used the 19 climatic variables and a digital elevation model available in WorldClim [**see** **Supporting Information—Table S2**; Hijmans *et al*. 2005; http://www.worldclim.org/bioclim.htm]. All variables had a spatial resolution of 1 km^2^. The distribution models were transformed into binary presence–absence maps using a 10 % omission error to determine the cut-off using ArcMap from ArcGIS (Environmental Systems Research Institute, Inc., New York).

**Figure 1.**
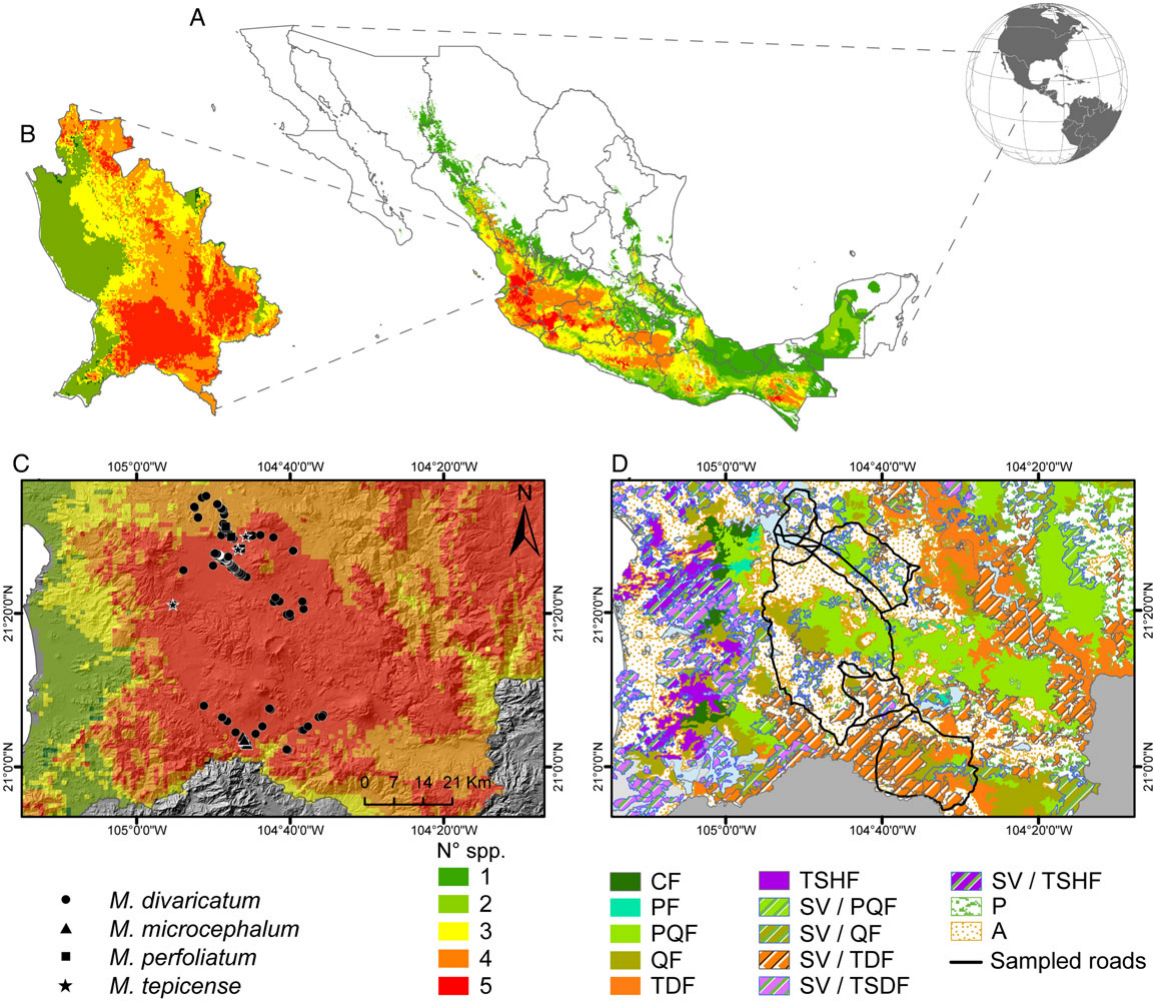
Overlapping models of the potential distribution of five species of *Melampodium* in (A) Mexico, (B) Nayarit and (C) the surveyed area. The colours indicate the probability of finding one (dark green) to five (red) species. The symbols show the sites of the populations of the four documented species in the surveyed area. Map (D) shows the vegetation types of the surveyed area and was adapted from INEGI (2013); it also shows the roads along which the survey was conducted. The abbreviations for the vegetation types are as follows: CF, cloud forest or tropical humid mountain forest; PF, pine (*Pinus*) forest; PQF, pine-oak forest; QF, oak (*Quercus*) forest; TDF, tropical dry (or deciduous) forest; TSHF, tropical subhumid forest; SV/PQF, secondary vegetation derived from pine-oak forest; SV/QF, secondary vegetation derived from oak forest; SV/TDF, secondary vegetation derived from tropical dry forest; SV/TSDF, secondary vegetation derived from tropical semi-dry (or semi-deciduous) forest; SV/TSHF, secondary vegetation derived from tropical subhumid forest; P, induced grassland; A, agricultural land.

Because field surveys are time consuming and expensive, we limited ours to an area of high predicted *Melampodium* diversity in Nayarit. To identify such an area, we first overlaid the presence–absence maps of all our focal *Melampodium* species for all of Mexico and identified areas where finding the five species was highly probable. Our species distribution approach yielded two main areas of high predicted diversity (Fig. 1). The largest one, on the western side of the Trans-Mexican Volcanic Belt, included the states of Nayarit, Jalisco, Colima and Michoacán. The second, smaller area was located in the southern Sierra Madre del Sur, which extends towards the Sierra Madre of Chiapas. We selected the polygon with the highest predicted diversity in the state of Nayarit as the area for our field surveys. This polygon had an area of 3174 km^2^ and was located within a region called the ‘Altiplanicie Nayarita’ (Anguiano 1992) that includes the main volcanos and intermontane valleys of the Trans-Mexican Volcanic Belt in the state.

The natural vegetation in the selected polygon consists of mixed montane oak and pine-oak forests, including some cloud forest in protected locations. There are some disturbed relicts of semi-evergreen tropical forests on the lower slopes, especially those facing north or west, and tropical dry forests cover the valleys in the eastern and southern portions (Fig. 1D). Because of their highly fertile soils, most of the valley area and foothills have a long history of agricultural use.

### Field data

We surveyed the area selected from our modelling approach (3174 km^2^) along the primary and secondary roads shown in Fig. 1D, from August to October 2012, the main flowering season. On 15 field days, all of the roads were slowly travelled three times at intervals. As the roads generally run along valleys, the populations on the hillsides with their bright yellow flowers are easily located from the roads. We visited all visible populations as well as sites that, based on previous information, were likely to host populations of *Melampodium*. In places with natural vegetation (see Fig. 1D) and in cultivated areas, we stopped and walked further from the road (a few hundred metres) to find populations. For every population found, we recorded species, habitat type and coordinates.

The data of our field survey were comparable with the data obtained from herbarium specimens, even though they were collected specifically along roads. A map of the processed herbarium specimens of *Melampodium* from central Mexico **[see** **Supporting Information—Fig. S1****]** shows clearly that few of these collections were made away from roads.

### Synanthropy index

To assess the plant's weediness, or ability to grow in habitats with varying degrees of association with anthropogenic environments (Hart 1976), we used a SI that takes into account three levels of disturbance: low (natural vegetation), intermediate (ruderal vegetation) and high (agrestal vegetation). We defined our SI as follows: SI = 3*x* + 2*y* + *z*, where *x*, *y* and *z* correspond to the fraction of the total number of individuals of a given species collected in agrestal, ruderal and natural sites, respectively. This newly defined index ranges from 1 to 3, with 3 representing the maximum association with habitats transformed by human activity.

Our SI is based on Nuorteva's index (1963), which is widely used to evaluate degree of insect association with urban, rural or natural habitats (Bueno Marí and Jiménez-Peydró 2011; Barata *et al*. 2012; Beltran *et al*. 2012; De Souza and Zuben 2012; Ekanem *et al*. 2013; Yepes-Gaurisas *et al*. 2013). Nuorteva's original index is calculated with the formula SI_Nuorteva_ = (1/2)(2*a* + *b* − 2*c*), where *a*, *b* and *c* are percentages of collections or captures in urban, rural and natural environments, respectively; values range from −100 to +100. This index is based on percentages, and the categories are weighted differentially. In contrast, our SI is based on proportions. As we considered ruderal vegetation, particularly in rural areas, to be intermediate between agrestals and natural vegetation, we gave it an intermediate weight.

### Statistical analyses

We first assessed the association between species and habitat category with a *χ*^2^ test. Then, to rule out the possibility that the observed association of a given species with a particular habitat type was the result of stochastic processes or sampling error, we conducted randomization tests. For these tests, we used only the data derived from herbarium records. The reason for excluding the field data was that the number of records was insufficient for most species, except *M. divaricatum*.

We compared the observed value for each habitat of a given species against a null distribution consisting of 1000 random samples as follows: from the original pool of 543 records, by sampling with replacement, we generated a distribution of 1000 datasets of a size equal to the number of accessions for a given species (i.e. for *M. americanum*, we sampled 79 records 1000 times; for *M. divaricatum*, 279; for *M. microcephalum*, 78; for *M. perfoliatum*, 92 and for *M. tepicence*, 15). We then tallied the habitat occurrence per sample (i.e. how many records per sample belong to each habitat category), thus generating null distributions for each habitat type per species. For every species, we compared the observed value for a specific habitat category against the null distributions for the habitat categories. If the observed values of habitat categories for the different species were merely an effect of sampling, we would expect that the observed value for all species/habitat combinations to fall within the 95 % confidence intervals (CI) of their respective resampling distributions. If, however, our observed associations between species and habitats reflect biological associations and not sampling error, we would expect the observed values to fall outside the 95 % CI of the resampling distributions of species–habitat combinations.

Over 50 % of the total records belonged to *M. divaricatum*. To investigate the extent to which the observed patterns were driven by this species, we repeated our resampling analyses excluding the records of *M. divaricatum*.

We also explored the possibility that the SI values were influenced by sample size. For this, we generated another set of null distributions (size 1000 again) for each species, this time resampling only 25, 50 or 75 % of the data, prior to re-calculating the SI for each species. Again, if SI calculations were independent of sample size, we would expect to obtain the same relative ranking for each of the species. For computational reasons, we calculated only 100 SI. All resampling analyses were done in R (R Development Core Team 2014; http/www.r-project.org/; code available upon request).

## Results

### Herbarium data

Four species were found in all three types of habitat (agrestal, ruderal and natural), in varying proportions (Table 1). The fifth species, *M. tepicense*, has not been reported as an agrestal weed in the literature, and we did not find any specimens from cultivated fields either. More than half of the specimens belonged to *M. divaricatum* and only 3 % to *M. tepicense*. The frequencies of the other three species were similar (*M. perfoliatum*: 17 %, *M. americanum* and *M. microcephalum*: 14 % each). Most specimens had been collected in natural vegetation (45 %), 41 % in ruderal habitats and only 14 % as agrestal plants.

**Table 1.** Number of records of the five focal species of *Melampodium* by habitat, obtained from the specimen and field survey data. The proportion (%) is in parentheses.

Type of data	Species	Agrestal	Ruderal	Natural	Total
Herbarium data	*M. americanum*	4 (5)	43 (54)	32 (41)	79
	*M. divaricatum*	47 (17)	118 (42)	114 (41)	279
	*M. microcephalum*	10 (13)	26 (33)	42 (54)	78
	*M. perfoliatum*	13 (14)	36 (39)	43 (47)	92
	*M. tepicense*	0 (0)	2 (13)	13 (87)	15
	Total	74	225	244	543
Field data	*M. americanum*	0 (0)	0 (0)	0 (0)	0
	*M. divaricatum*	9 (6)	140 (94)	0 (0)	149
	*M. microcephalum*	0 (0)	10 (71)	4 (29)	14
	*M. perfoliatum*	0 (0)	7 (100)	0 (0)	7
	*M. tepicense*	0 (0)	2 (67)	1 (33)	3
	Total	9	159	5	173

### Field data

We found 173 populations of four of the five species in the area predicted by models based on climatic data (Table 1). We were not able to locate populations of *M. americanum*; this species has its northern limit in the state and is known from only two collections there. In contrast to what herbarium data would suggest, most populations were ruderal (92 %), 5 % were agrestal and 3 % grew in natural vegetation. The only species found in a variety of cultivated fields and plantations (including maize, avocado, roselle–*Hibiscus sabdariffa*, green beans and lime) was *M. divaricatum*. In contrast, *M. microcephalum* and *M. tepicense* were mostly found in tropical dry forest and semi-evergreen tropical forest, respectively. As with herbarium data, most of the populations belonged to *M. divaricatum* (86 %) and very few to *M. tepicense* (2 %), although *M. microcephalum* and *M. perfoliatum* were also low in numbers (8 and 4 %, respectively).

### Synanthropy index and species–habitat associations

Species of *Melampodium* varied in their degree of weediness, as was reflected by their SI values (Table 2). As expected, *M. divaricatum* had the highest SI values (SI_FIELD_ = 2.06, SI_HERBARIUM_ = 1.76), and *M. tepicense* the lowest (SI_FIELD_ = 1.67, SI_HERBARIUM_ = 1.13). Values of SI obtained from field data tended to be higher than those derived from herbarium data, but the relative ranking of species was conserved.

**Table 2.** Synanthropy index values for the studied *Melampodium* species using herbarium specimens and field surveys.

Species	SI	
	Specimen data	Field data
*M. divaricatum*	1.76	2.06
*M. perfoliatum*	1.67	2.00
*M. americanum*	1.64	–
*M. microcephalum*	1.59	1.71
*M. tepicense*	1.13	1.67

**Figure 2.**
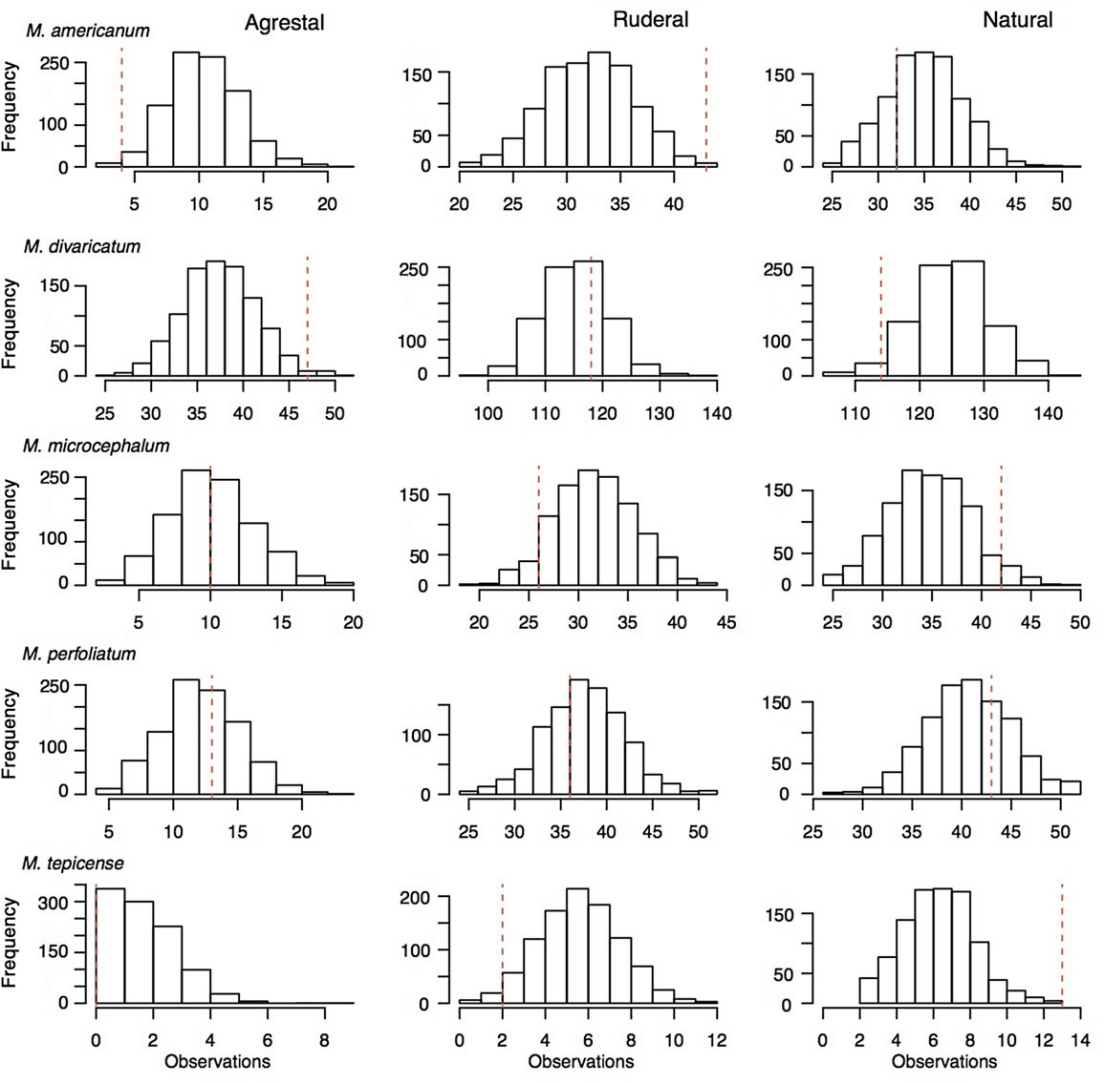
Observed values (red line) and histograms of the distributions of the specimen data from agrestal, ruderal and natural habitats, after 1000 random resamplings for each species.

A *χ*^2^ independence test for both the herbarium $\left(\chi^{2}=24.59>\chi_{0.05,8}^{2}=15.51\right)$ and the field data $\left(\chi^{2}=48.49>\chi_{0.05,6}^{2}=12.59\right)$ suggested that the species differed in the kind of habitat they occupy. We infer that this relationship probably reflects a biological and not a stochastic phenomenon, as most of the observed SI values, with the exception of those for *M. microcephalum* and *M. perfoliatum* as agrestals, fell outside the 95 % CI derived from randomized datasets (Table 3, Fig. 2).

**Table 3.** Observed records and expected values (median and 95 % CI, *α* = 0.05, df = 999) for each species and habitat combination under randomized data. Values that fell outside the 95 % CI and are thus interpreted to be significantly different from random are highlighted in bold. Species abbreviations are as follows: Mam, *Melampodium americanum*; Mdi, *M. divaricatum*; Mmi, *M. microcephalum*; Mpe, *M. perfoliatum*; Mte, *M. tepicense*.

Species	Agrestal habitat			Ruderal habitat			Natural vegetation		
	Observed records	Expected median	Expected 95 % CI	Observed records	Expected median	Expected 95 % CI	Observed records	Expected median	Expected 95 % CI
Mam	**4**	11	10.3, 11.5	**43**	32.6	31.7, 33.5	**32**	35.5	34.6, 36.4
Mdi	**47**	38	37.5, 38.5	**118**	115.6	114.9, 116.3	**114**	125.4	124.7, 126.1
Mmi	10	11	9.9, 11.3	**26**	32.1	31.2, 33.0	**42**	35.3	34.2, 36.2
Mpe	13	13	12.0, 13.2	**36**	38.1	37.2, 39.0	**43**	41.3	40.4, 42.1
Mte	**0**	2	1.5, 2.7	**2**	6.1	5.1, 7.1	**13**	6.8	5.8, 7.8

Excluding *M. divaricatum* from our analyses did not shift patterns in species' null distributions **[see** **Supporting Information—Diagram S1****]**. These results suggest that the patterns we observed were not driven by *M. divaricatum*, the species with most herbarium records and populations. The only exception was *M. perfoliatum*, which appeared to be slightly weedier when we excluded *M. divaricatum*.

**Figure 3.**
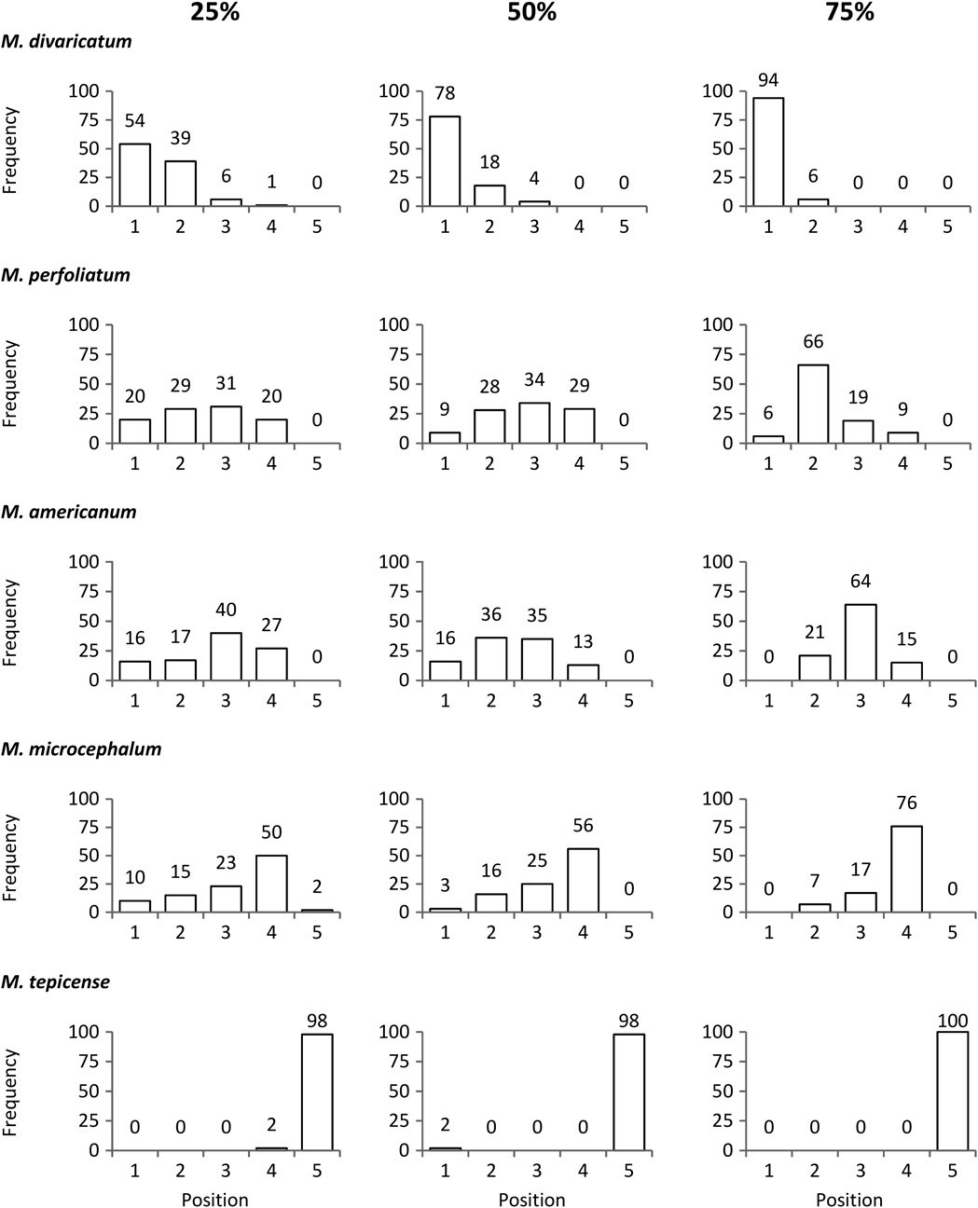
Frequency of relative rankings by species based on SI, after 100 random bootstrap resamplings with 25, 50 and 75 % of all the species records. On the *x*-axis, the number 1 indicates the first position in agreement with the SI of the other species, while the number 5 indicates the last position.

The species rankings were robust against a reduction of the sample size, as their relative ranks were maintained when randomly resampling from subsequently smaller datasets. *Melampodium divaricatum*, *M. microcephalum* and *M. tepicense* preserved their first, fourth and fifth places, respectively (Fig. 3); *M. perfoliatum* and *M. americanum* occupied intermediate ranks, sometimes interchanging second and third place.

## Discussion

Our field validation of the relative weediness of the species confirmed, with slight variations, the ranking initially calculated from herbarium data (and the usefulness of distribution modelling). Thus, our results suggest that herbarium data are an appropriate resource for ranking species by their degree of association with human activities, as reflected by synanthropy indices. This is further evidence (Lavoie 2013) that biological collections are important tools for obtaining ecological data.

This is the first time herbarium and field data for weed habitat are compared directly and quantitatively, and one of the few comparisons for any group of plants. The only other example we found, Garcillán and Ezcurra (2011), evaluated herbarium and direct field data from vegetation plots for an island, in order to calculate their rarefaction curves (and coverage of rare species). They found that collections over-represent rare species, but due to the same collection bias, also deliver more complete species lists.

Their results are compatible with ours: the herbarium data yielded consistently lower SI values than field data. *Melampodium divaricatum*, the most widespread and abundant species (Turner and King 1962; Stuessy 1979), represented 51 % of the herbarium specimens, but 86 % of the populations found in the field. This confirms previous findings that collectors do not collect common species in proportion to their presence (Guralnick and Van Cleve 2005; Garcillán and Ezcurra 2011). The data also agree with previous observations suggesting that biologists tend to collect in easily accessible places with natural vegetation and often avoid secondary vegetation (Rich and Woodruff 1992; Kadmon *et al*. 2004; Pyke and Ehrlich 2010; Kramer-Schadt *et al*. 2013).

Our field observations indicated that *Melampodium* species were basically ruderal. However, label data from herbarium specimens often lack data on the microhabitat where plants really grow. Misrepresentation of habitat in our data remains a possibility. For example, a label may indicate a forested area, but the plants might have been actually growing on the side of the path or in a clearing, in a more ruderal setting. However, we have no reason to suspect that such biases affected species differentially in a systematic way.

A shortcoming of studies based on herbarium or database records is the potential for misidentifications and errors in the label information. In our case, these sources of error should be low, as we put considerable effort into curating both collections and database information, the taxonomy and phylogeny of *Melampodium* are well known and all specimens had been recently examined and annotated by a specialist in the genus or family. However, this should be kept in mind when using uncorroborated data from, for example, public databases.

As a final note, we would like to point out that the relative ranking of *Melampodium* species based on their SI values seems to correspond to the size of the species' geographical range. According to Stuessy (1979), the six weediest taxa of the genus in order of their distribution size are, in descending order, *M. divaricatum* (SI_HERBARIUM_ = 1.76), the weediest and most widely distributed species, which has been recorded in 33 administrative units (states for Mexico and countries for Central America), *M. perfoliatum* (SI_HERBARIUM_ = 1.67) known from 19 administrative units, *M. sericeum* from 18, *M. gracile* from 14, *M. americanum* (SI_HERBARIUM_ = 1.64) and *M. linearilobum* from 12 each. The two other species of our study are present in 11 administrative units (*M. microcephalum*, SI_HERBARIUM_ = 1.59) and 4 administrative units (*M. tepicense*, SI_HERBARIUM_ = 1.13). *Melampodium tepicense* is the least weedy taxon and is restricted to pine-oak forests, cloud forests and tropical dry forests in the Sierra de San Juan (Nayarit), Sierra de Manantlán (Jalisco), the Nevado de Colima and Coalcomán (Michoacán) (Stuessy 1972). Calculating SI for other taxa would shed light on the generality of this relationship (but see López-Sandoval 2014). The positive correlation between traits associated with weediness (e.g. the annual life span and the long flowering period or the germination niche breadth) and range size has also been documented for weeds in Europe (Brändle *et al*. 2003; Lososová *et al*. 2008), as has the relationship between some other features characterizing weediness (e.g. early and extended flowering, in some cases, the annual life span; the ‘general purpose genotype’) and invasiveness (Rejmánek 2000; Pyšek and Richardson 2007).

## Conclusions

Herbarium specimens reflect the weediness of species relative to each other. Calculating an index from herbarium or database records is an economical first step for identifying weeds or species with weedy characteristics and ranking them in relative order, which is useful for comparative studies on the biology and ecology of a group of plants. However, collection data do not lead to absolute rankings of weediness or synanthropy, due to collection bias. Detailed field studies are necessary for obtaining definite values for this ecological trait. Finally, our work also shows the utility of species distribution modelling as a tool for guiding field surveys.

## Sources of Funding

The work of the first author was supported by the CONACyT (Mexico) project NAYARIT-2008-C 01-93389 (‘Fortalecimiento y consolidación del Doctorado en Ciencias Biológicas y Agropecuarias con énfasis en el área de ciencias ambientales de la Universidad Autónoma de Nayarit’).

## Contributions by the Authors

The work was conceived by A.M.H.-A., H.V. and J.L.V.; A.M.H.-A. did the field and herbarium work, and wrote the first version in Spanish; J.L.V. created and curated the Asteraceae database, and verified specimen identifications; A.M.H.-A., J.L.V., N.I.C., E.O. and V.A.G.-G. did the modelling and statistical analysis and H.V. wrote the English version, which was then revised and commented on by all authors.

## Conflict of Interest Statement

None declared.

## Supporting Information

The following additional information is available in the online version of this article –

**Table S1.** General distribution, habitat and phenology of the five species of *Melampodium* selected for this study, based on Stuessy (1972) and Robinson (1901).

**Table S2.** Environmental variables used as predictors of the model of the potential distribution.

**Figure S1.** A map of the general collections of the five *Melampodium* species of this study, previous to this work, with major roads. It shows that the large majority of the collections were made along roadsides.

**Diagram S1.** Histograms of 1000 null distributions generated by resampling the specimen data in R (Core Development Team), and excluding *Melampodium divaricatum*.

Additional Information

## References

[PLV144C1] Anguiano M 1992 Nayarit costa y altiplanicie en el momento del contacto. México DF: Universidad Nacional Autónoma de México.

[PLV144C2] Baker HG 1965 Characteristics and modes of origin of weeds. In: Baker HG, Stebbins GL, eds. The genetics of colonizing species. New York: Academic Press, 147–168.

[PLV144C3] Barata RA, Ursine RL, Nunes FP, Morais DH, Araújo HS 2012 Synanthropy of mosquitoes and sand flies near the Aimorés hydroelectric power plant, Brazil. Journal of Vector Ecology 37:397–401. 10.1111/j.1948-7134.2012.00243.x 23181864

[PLV144C4] Beltran YTP, Segura NA, Bello FJ 2012 Synanthropy of Calliphoridae and Sarcophagidae (Diptera) in Bogotá, Colombia. Neotropical Entomology 41:237–242. 10.1007/s13744-012-0036-x 23950049

[PLV144C5] Blöch C 2010 *Molecular phylogeny and chromosome evolution of the genus* Melampodium *L. (Millerieae, Asteraceae)*. Doctoral Thesis, Vienna University, Vienna.

[PLV144C6] Blöch C, Weiss-Schneeweiss H, Schneeweiss GM, Barfuss MHJ, Rebernig CA, Villaseñor JL, Stuessy TF 2009 Molecular phylogenetic analyses of nuclear and plastid DNA sequences support dysploid and polyploid chromosome number changes and reticulate evolution in the diversification of *Melampodium* (Millerieae, Asteraceae). Molecular Phylogenetics and Evolution 53:220–233. 10.1016/j.ympev.2009.02.021 19272456 PMC4268500

[PLV144C7] Bolmgren K, Lönnberg K 2005 Herbarium data reveal an association between fleshy fruit type and earlier flowering time. International Journal of Plant Sciences 166:663–670. 10.1086/430097

[PLV144C8] Brändle M, Stadler J, Klotz S, Brandl R 2003 Distributional range size of weedy plant species is correlated to germination patterns. Ecology 84:136–144. 10.1890/0012-9658(2003)084[0136:DRSOWP]2.0.CO;2

[PLV144C9] Bueno Marí R, Jiménez-Peydró R 2011 Differences in mosquito (Diptera: Culicidae) biodiversity across varying climates and land-use categories in Eastern Spain. Entomologica Fennica 22:190–198.

[PLV144C10] Crawford PHC, Hoagland BW 2009 Can herbarium records be used to map alien species invasion and native species expansion over the past 100 years? Journal of Biogeography 36:651–661. 10.1111/j.1365-2699.2008.02043.x

[PLV144C11] De Souza CR, Zuben CJV 2012 Diversity and synanthropy of Calliphoridae (Diptera) in the region of Rio Claro, SP, Brazil. Neotropical Entomology 41:243–248. 10.1007/s13744-012-0037-9 23950050

[PLV144C12] Ekanem MS, Idiong MO, Usua EJ 2013 Synanthropic indices and baits preferences of common non-biting flies (Diptera: Cyclorrhapha) of Akwa Ibom State, Nigeria. International Journal of Biodiversity and Conservation 5:192–197.

[PLV144C13] Garcillán PP, Ezcurra E 2011 Sampling procedures and species estimation: testing the effectiveness of herbarium data against vegetation sampling in an oceanic island. Journal of Vegetation Science 22:273–280. 10.1111/j.1654-1103.2010.01247.x

[PLV144C14] Gil GE, Lobo JM 2012 El uso de modelos predictivos de distribución para el diseño de muestreos de especies poco conocidas. Mastozoología Neotropical 19:47–62.

[PLV144C15] Graham CH, Ferrier S, Huettman F, Moritz C, Peterson AT 2004 New developments in museum-based informatics and applications in biodiversity analysis. Trends in Ecology and Evolution 19:497–503. 10.1016/j.tree.2004.07.006 16701313

[PLV144C16] Guralnick R, Van Cleve J 2005 Strengths and weaknesses of museum and national survey data sets for predicting regional species richness: comparative and combined approaches. Diversity and Distributions 11:349–359. 10.1111/j.1366-9516.2005.00164.x

[PLV144C17] Hart R 1976 An index for comparing weediness in plants. Taxon 25:245–247. 10.2307/1219447

[PLV144C18] Hijmans RJ, Cameron SE, Parra JL, Jones PG, Jarvis A 2005 Very high resolution interpolated climate surfaces for global land areas. International Journal of Climatology 25:1965–1978. 10.1002/joc.1276

[PLV144C19] Hill MO, Roy DB, Thompson K 2002 Hemeroby, urbanity and ruderality: bioindicators of disturbance and human impact. Journal of Applied Ecology 39:708–720. 10.1046/j.1365-2664.2002.00746.x

[PLV144C20] Holzner W 1978 Weed species and weed communities. Vegetatio 38:13–20. 10.1007/BF00141295

[PLV144C21] Holzner W 1982 Concepts, categories and characteristics of weeds. In: Holzner W, Numata M, eds. Biology and ecology of weeds. Den Haag: Dr W. Junk Publishers, 3–20.

[PLV144C22] INEGI. 2013 Conjunto de datos vectoriales de uso del suelo y vegetación. Serie V, escala 1:250000. Aguascalientes, México: Instituto Nacional de Estadística, Geografía e Informática.

[PLV144C23] Kadmon R, Farber O, Danin A 2004 Effect of roadside bias on the accuracy of predictive maps produced by bioclimatic models. Ecological Applications 14:401–413. 10.1890/02-5364

[PLV144C24] Klotz S, Kühn I 2002 Indikatoren des anthropogenen Einflusses auf die Vegetation [Indicators of anthropogenic influence on the vegetation]. Schriftenreihe für Vegetationskunde 38:241–246.

[PLV144C25] Kramer-Schadt S, Niedballa J, Pilgrim JD, Schröder B, Lindenborn J, Reinfelder V, Stillfried M, Heckmann I, Scharf AK, Augeri DM, Cheyne SM, Hearn AJ, Ross J, Macdonald DW, Mathai J, Eaton J, Marshall AJ, Semiadi G, Rustam R, Bernard H, Alfred R, Samejima H, Duckworth JW, Breitenmoser-Wuersten C, Belant JL, Hofer H, Wilting A 2013 The importance of correcting for sampling bias in MaxEnt species distribution models. Diversity and Distributions 19:1366–1379. 10.1111/ddi.12096

[PLV144C26] Kuester A, Conner JK, Culley T, Baucom RS 2014 How weeds emerge: a taxonomic and trait-based examination using United States data. New Phytologist 202:1055–1068. 10.1111/nph.12698 24494694 PMC4235316

[PLV144C27] Lavoie C 2013 Biological collections in an ever changing world: herbaria as tools for biogeographical and environmental studies. Perspectives in Plant Ecology, Evolution and Systematics 15:68–76. 10.1016/j.ppees.2012.10.002

[PLV144C28] López-Sandoval JA 2014 Índice de sinantropía, crecimiento y distribución de especies del género Physalis, subgénero Rydbergis, sección Angulatae. PhD Thesis, Colegio de Postgraduados, México.

[PLV144C29] Lososová Z, Chytrý M, Kühn I 2008 Plant attributes determining the regional abundance of weeds on central European arable land. Journal of Biogeography 35:177–187.

[PLV144C30] Mateo RG, Felicísimo ÁM, Muñoz J 2011 Modelos de distribución de especies: Una revisión sintética. Revista Chilena de Historia Natural 84:217–240. 10.4067/S0716-078X2011000200008

[PLV144C31] Mcvaugh R 1984 Compositae, Flora Novo-Galiciana. A descriptive account of the vascular plants of western Mexico, Vol. 12 Ann Arbor: University of Michigan Press.

[PLV144C32] Nuorteva P 1963 Synanthropy of blowflies (Diptera, Calliphoridae) in Finland. Annales Entomologica Fennica 29:1–49.

[PLV144C33] Ortiz BE, Villaseñor JL, Téllez O 1998 La familia Asteraceae en el estado de Nayarit (México). Acta Botanica Mexicana 44:25–57.

[PLV144C34] Phillips SJ, Anderson RP, Schapire RE 2006 Maximum entropy modeling of species geographic distributions. Ecological Modelling 190:231–259. 10.1016/j.ecolmodel.2005.03.026

[PLV144C35] Pyke GH, Ehrlich PR 2010 Biological collections and ecological/environmental research: a review, some observations and a look to the future. Biological Reviews 85:247–266. 10.1111/j.1469-185X.2009.00098.x 19961469

[PLV144C36] Pyšek P, Richardson DM 2007 Traits associated with invasiveness in alien plants: where do we stand? In: Nentwig W, ed. Biological invasions. Heidelberg: Springer, 97–125.

[PLV144C36a] R Development Core Team. 2014 R: A language and environment for statistical computing. Vienna, Austria: The R Foundation for Statistical Computing http://www.r-project.org/.

[PLV144C37] Radosevich S, Holt J, Ghersa C 1997 Weed ecology. Implications for management, 2nd edn New York: Wiley.

[PLV144C38] Rejmánek M 2000 Invasive plants: approaches and predictions. Austral Ecology 25:497–506. 10.1046/j.1442-9993.2000.01080.x

[PLV144C39] Rich T, Woodruff E 1992 Recording bias in botanical surveys. Watsonia 19:73–95.

[PLV144C40] Robbirt KM, Davy AJ, Hutchings MJ, Roberts DL 2011 Validation of biological collections as a source of phenological data for use in climate change studies: a case study with the orchid *Ophrys sphegodes*. Journal of Ecology 99:235–241. 10.1111/j.1365-2745.2010.01727.x

[PLV144C41] Robinson BL 1901 Synopsis of the genus *Melampodium*. Proceedings of the American Academy of Arts and Sciences 36:455–466. 10.2307/20021597

[PLV144C42] Rzedowski J 2006 Vegetación de México, 1st digital edn México DF: Comisión Nacional para el Conocimiento y Uso de la Biodiversidad.

[PLV144C43] Šilc U 2010 Synanthropic vegetation: pattern of various disturbances on life history traits. Acta Botanica Croatica 69:215–227.

[PLV144C44] Stuessy TF 1971 Chromosome numbers and phylogeny in *Melampodium* (Compositae). American Journal of Botany 58:732–736. 10.2307/2441471

[PLV144C45] Stuessy TF 1972 Revision of the genus *Melampodium* (Compositae: Heliantheae). Rhodora 74:1–70, 161–219.

[PLV144C46] Stuessy TF 1979 Cladistics of *Melampodium* (Compositae). Taxon 28:179–195. 10.2307/1219575

[PLV144C47] Stuessy TF, Weiss-Schneeweiss H, Keil DJ 2004 Diploid and polyploid cytotype distribution in *Melampodium cinereum* and *M. leucanthum* (Asteraceae, Heliantheae). American Journal of Botany 91:889–898. 10.3732/ajb.91.6.889 21653445

[PLV144C48] Téllez O 1995 Flora, vegetación y fitogeografía de Nayarit, México. Master's Thesis, Facultad de Ciencias, Universidad Nacional Autónoma de México, México DF.

[PLV144C49] Turner BL, King RM 1962 A cytotaxonomic survey of *Melampodium* (Compositae-Heliantheae). American Journal of Botany 49:263–269. 10.2307/2439548

[PLV144C50] Vieyra-Odilon L, Vibrans H 2001 Weeds as crops: the value of maize field weeds in the Valley of Toluca, Mexico. Economic Botany 55:426–443. 10.1007/BF02866564

[PLV144C51] Villaseñor JL 2003 Diversidad y distribución de las Magnoliophyta de México. Interciencia 28:160–167.

[PLV144C52] Villaseñor JL, Espinosa-García FJ 1988 Catálogo de las malezas de México. México DF: Universidad Nacional Autónoma de México y Fondo de Cultura Económica.

[PLV144C53] Wegier A, Piñeyro-Nelson A, Alarcón J, Gálvez-Mariscal A, Álvarez-Buylla ER, Piñero D 2011 Recent long-distance transgene flow into wild populations conforms to historical patterns of gene flow in cotton (*Gossypium hirsutum*) at its centre of origin. Molecular Ecology 20:4182–4194. 10.1111/j.1365-294X.2011.05258.x 21899621

[PLV144C54] Weiss-Schneeweiss H, Villaseñor JL, Stuessy TF 2009 Chromosome numbers, karyotypes, and evolution in *Melampodium* (Asteraceae). International Journal of Plant Sciences 170:1168–1182. 10.1086/605876

[PLV144C55] Wu SH, Rejmánek M, Grotkopp E, Ditomaso JM 2005 Herbarium records, actual distribution, and critical attributes of invasive plants: genus *Crotalaria* in Taiwan. Taxon 54:133–138. 10.2307/25065311

[PLV144C56] Yepes-Gaurisas D, Sánchez-Rodríguez JD, de Mello-Patiu CA, Echeverri MW 2013 Synanthropy of Sarcophagidae (Diptera) in La Pintada, Antioquia-Colombia. Revista de Biología Tropical 61:1275–1287. 10.15517/rbt.v61i3.11955 24027923

