# Supplementary material for: Use of herbarium data to evaluate weediness in five congeners
Source: AoB Plants. 2015 Dec 15;8:plv144. doi: 10.1093/aobpla/plv144 (PMC4740360; doi:10.1093/aobpla/plv144)
Supplement: Additional Information [file supp_8_plv144_index.html]

Use of herbarium data to evaluate weediness in five congeners — Use of herbarium data to evaluate weediness in five congeners — Additional Information 

# Use of herbarium data to evaluate weediness in five congeners

## Additional Information

Additional Information

- Supplementary file1 - doc file
- Supplementary file2 - doc file
- Supplementary file3 - doc file
- Supplementary file4 - doc file
