# Supplementary material for: Use of herbarium data to evaluate weediness in five congeners
Source: AoB Plants. 2015 Dec 15;8:plv144. doi: 10.1093/aobpla/plv144 (PMC4740360; doi:10.1093/aobpla/plv144)
Supplement: Additional Information [file supp_plv144_plv144supp_file1.doc]

**Supporting information 1** for the paper

Hanan-A., A.M. et al. Use of herbarium data to evaluate weediness in five congeners. *AoB PLANTS*.

**S1.** General distribution, habitat and phenology of the five species of *Melampodium* selected for this study, based on Stuessy (1972) and Robinson (1901).

| **Species**  Habitat and distribution | **Elevation (m)** | **Flowering period** |
| --- | --- | --- |
| ***Melampodium americanum* L.**  Grows from tropical grasslands to pine-oak forests; in Mexico on the eastern slopes of the eastern Sierra Madre and the western slopes of the western and southern Sierra Madre, extending to Guatemala | 210 - 2380 | January to August |
| ***Melampodium divaricatum* (Rich.) DC.**  Mainly in the subtropics, many habitats; from Mexico to Central America and northeastern Colombia, also in eastern Brazil; introduced in Cuba, Burma, Puerto Rico and the Virgin Islands | 15 - 2990 | Year-round |
| ***Melampodium microcephalum* Less.**  In pine-oak forests, tropical dry and humid forests; in the central highlands and the western and southern Sierra Madre in Mexico, also in Guatemala | 50 - 1740 | June to November |
| ***Melampodium perfoliatum* (Cav.) Kunth**  In tropical dry forests and pine-oak forests; in México, Guatemala, and Costa Rica, introduced in Cuba and southern California | 610-2440 | Year-round |
| ***Melampodium tepicense* B.L. Rob.**  In cloud forests, pine-oak forests and tropical dry forests; Nayarit, Jalisco, Colima and Michoacán | 100-1740 | August to February |
