# Supplementary material for: Use of herbarium data to evaluate weediness in five congeners
Source: AoB Plants. 2015 Dec 15;8:plv144. doi: 10.1093/aobpla/plv144 (PMC4740360; doi:10.1093/aobpla/plv144)
Supplement: Additional Information [file supp_plv144_plv144supp_file2.doc]

**Supporting information 2** for the paper

Hanan-A., A.M. et al. Use of herbarium data to evaluate weediness in five congeners. *AoB PLANTS*.

**S2.** Environmental variables used as predictors of the model of the potential distribution.

| **Variable** | |
| --- | --- |
| E | Elevation |
| BIO_1_ | Annual Mean Temperature |
| BIO_2_ | Mean Diurnal Range (Mean of monthly (max temp - min temp)) |
| BIO_3_ | Isothermality (BIO_2_/BIO_7_) (* 100) |
| BIO_4_ | Temperature Seasonality (standard deviation *100) |
| BIO_5_ | Max Temperature of Warmest Month |
| BIO_6_ | Min Temperature of Coldest Month |
| BIO_7_ | Temperature Annual Range (BIO_5_-BIO_6_) |
| BIO_8_ | Mean Temperature of Wettest Quarter |
| BIO_9_ | Mean Temperature of Driest Quarter |
| BIO_10_ | Mean Temperature of Warmest Quarter |
| BIO_11_ | Mean Temperature of Coldest Quarter |
| BIO_12_ | Annual Precipitation |
| BIO_13_ | Precipitation of Wettest Month |
| BIO_14_ | Precipitation of Driest Month |
| BIO_15_ | Precipitation Seasonality (Coefficient of Variation) |
| BIO_16_ | Precipitation of Wettest Quarter |
| BIO_17_ | Precipitation of Driest Quarter |
| BIO_18_ | Precipitation of Warmest Quarter |
| BIO_19_ | Precipitation of Coldest Quarter |
