# Supplementary material for: Use of herbarium data to evaluate weediness in five congeners
Source: AoB Plants. 2015 Dec 15;8:plv144. doi: 10.1093/aobpla/plv144 (PMC4740360; doi:10.1093/aobpla/plv144)

**Supporting information 3** for the paper

Hanan-A., A.M. et al. Use of herbarium data to evaluate weediness in five congeners. *AoB PLANTS.*

A map of the general collections of the five *Melampodium* species of this study, previous to this work, with major roads. It shows that the large majority of the collections were made along roadsides.


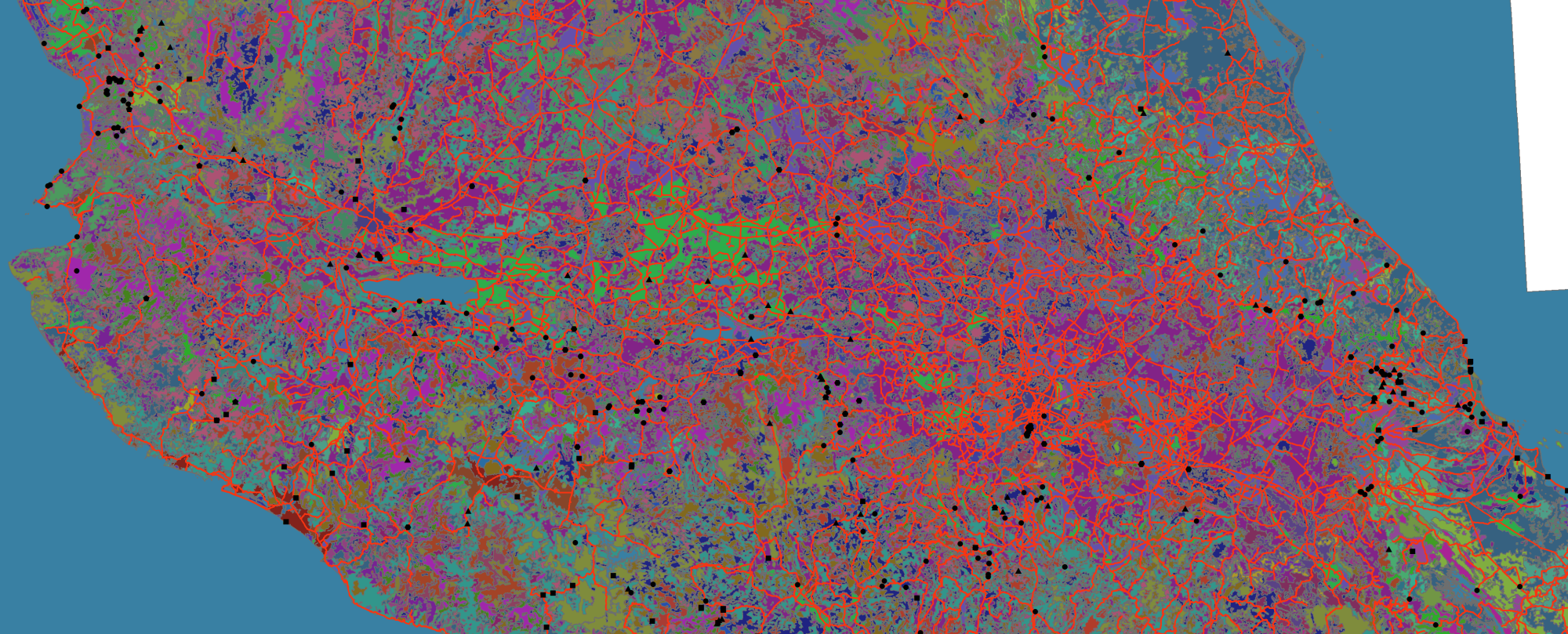

Supplement: Additional Information [file supp_plv144_plv144supp_file3.doc]
