# Supplementary material for: Use of herbarium data to evaluate weediness in five congeners
Source: AoB Plants. 2015 Dec 15;8:plv144. doi: 10.1093/aobpla/plv144 (PMC4740360; doi:10.1093/aobpla/plv144)

**Supporting information 4** for the paper

Hanan-A., A.M. et al. Use of herbarium data to evaluate weediness in five congeners. *AoB PLANTS*.

**S4.** Histograms of 1000 null distributions generated by resampling the specimen data in R (Core Development Team), and excluding *Melampodium divaricatum*. Observed values are indicated with a dotted red line, and null distributions by habitat preference (agrestal, ruderal and natural) derived from 1000 random resamplings for each species while excluding *M. divaricatum* from analyses (which accounts for >50% of records– distribution shown in second panel).


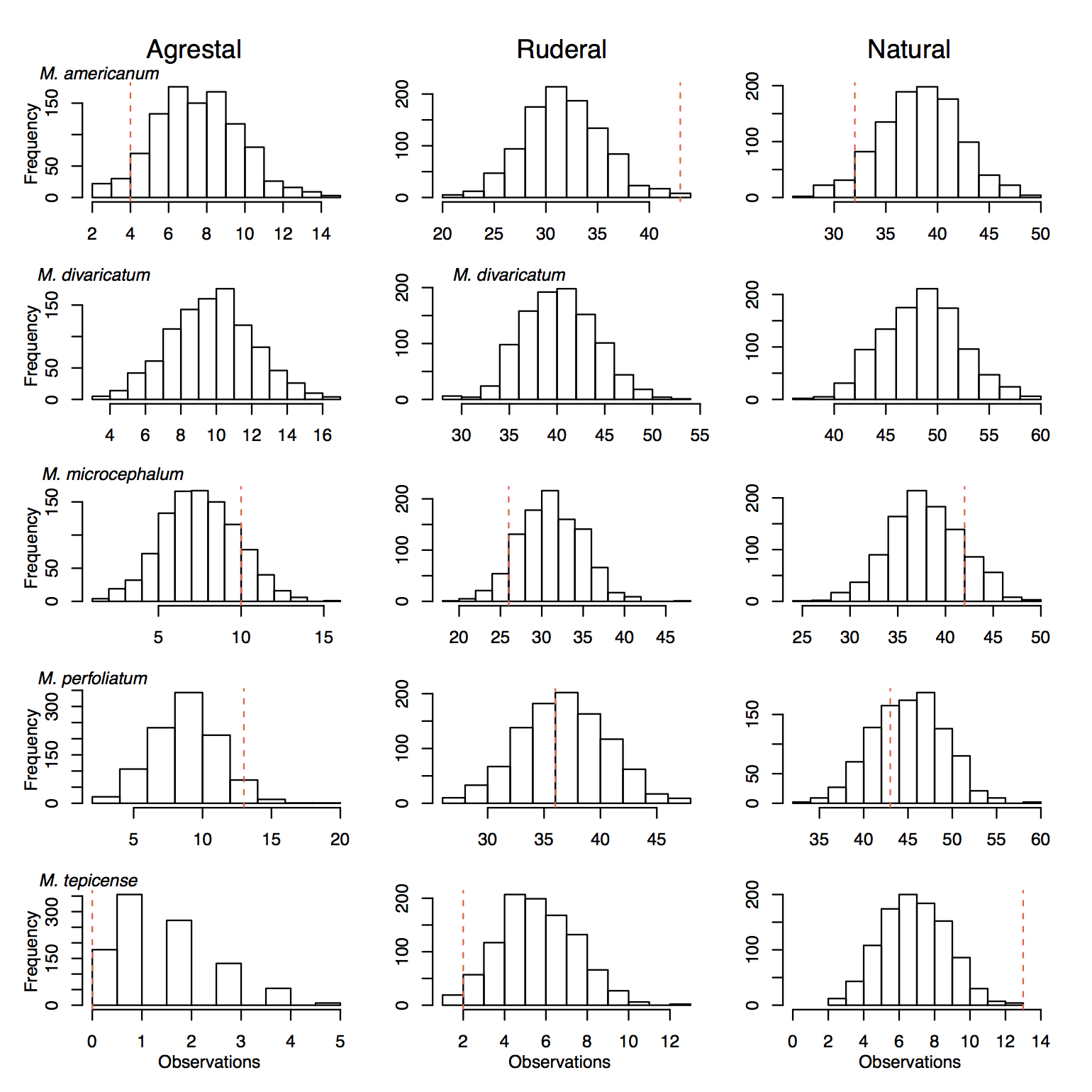

Supplement: Additional Information [file supp_plv144_plv144supp_file4.doc]
